# Longitudinal variance components models for systolic blood pressure, fitted using Gibbs sampling

**DOI:** 10.1186/1471-2156-4-S1-S25

**Published:** 2003-12-31

**Authors:** Katrina J Scurrah, Martin D Tobin, Paul R Burton

**Affiliations:** 1 Institute of Genetics and Department of Epidemiology and Public Health, University of Leicester, 22-28 Princess Road, Leicester, LE1 6TP, United Kingdom

## Abstract

This paper describes an analysis of systolic blood pressure (SBP) in the Genetic Analysis Workshop 13 (GAW13) simulated data. The main aim was to assess evidence for both general and specific genetic effects on the baseline blood pressure and on the rate of change (slope) of blood pressure with time. Generalized linear mixed models were fitted using Gibbs sampling in WinBUGS, and the additive polygenic random effects estimated using these models were then used as continuous phenotypes in a variance components linkage analysis. The first-stage analysis provided evidence for general genetic effects on both the baseline and slope of blood pressure, and the linkage analysis found evidence of several genes, again for both baseline and slope.

## Background

Longitudinal studies of families reduce measurement error, provide increased power, and allow researchers to assess genetic and environmental effects on both baseline measurements of phenotypes and the rate of change of the phenotype over time. However, care must be taken to use an appropriate model that fully accounts for both the intra-familial and the intra-individual correlation in the repeated measurements. This paper describes such a model, which is an extension of our previously described generalized linear mixed models (GLMMs) for cross sectional familial data [1,2], and applies this model to Replicate 1 of the Genetic Analysis Workshop 13 (GAW13) simulated data.

The aims of our analysis were three-fold: 1) to assess evidence for genetic effects on the rate of increase of SBP with age, as well as genetic effects on the blood pressure at any given time, 2) to determine which of the available covariates were associated with SBP, while correctly accounting for the complex familial correlation structure, and 3) to assess the performance of models for longitudinal familial data when applied to realistic data simulated by groups other than our own. Results of fitting GLMMs and variance components linkage models are presented and discussed.

## Methods

### Data

We analyzed the repeated measurements of systolic blood pressure (SBP) in the first replicate of the simulated GAW13 data with missing values, including all 4692 individuals in all 330 pedigrees. Analyses were done blind to the answers.

Each missing value of the covariates 'drink' (number of drinks per week) and 'CPD' (number of cigarettes per day) was replaced with the mean of the covariate for that individual in the two observations surrounding the missing observation, enabling us to increase the number of useful observations without excluding important covariates from the model. The phenotypic component of the model included 25,055 observations for 2851 individuals. These observations all had non-missing values for SBP, height, drink, and CPD.

### Model

The models fitted in this analysis are extensions of our previously described variance components models for extended pedigrees with normal, binary, or censored survival phenotypes [1,2]. The original models are GLMMs with components of variance due to additive polygenic effects (σ^2^_A_), common family environment (σ^2^_C_), common sibling environment (σ^2^_Cs_), and residual error (σ^2^_E_). The complex familial correlation structure is modelled through the use of shared random effects for each variance component. The model for the *j*^th ^individual in the *i*^th ^pedigree can be represented as:

μ_ij _= β_0 _+ β^T^X_ij _+ A_ij _+ C_ij _+ Cs_ij_

y_ij _~ N(μ_ij_, σ^2^_E_),

where y_ij _is the observed phenotype, X_ij _is a matrix of covariates,

**A_i _**~ N(**0**, V_A_), **C_i _**~ N(**0**, V_C_), **Cs_i _**~ N(**0**, V_Cs_),

and the random effects for each family are sampled from appropriate multivariate normal distributions with variance-covariance matrices (V_A_, V_C_, and V_Cs_) parameterized by σ^2^_A_, σ^2^_C_, and σ^2^_Cs_, respectively, and structured as described previously [1]. Individuals without phenotypic data were used in generating the random effects but not in the main model.

The extensions not only allow for repeated measures on individuals, but use the extra information available to assess evidence for genetic effects on the rate of change of the outcome over time. At the *k*^th ^observation for the *j*^th ^individual in the *i*^th ^pedigree, the model can be expressed as

μ_ijk _= β_0 _+ β^T^X_ijk _+ A_ij _+ C_ij _+ Cs_ij _+ age_ijk _× (β_age _+ A.time_ij _+ C.time_ij _+ Cs.time_ij _+ E.time_ij_)

y_ijk _~ N(μ_ijk_, σ^2^_E_),

where y_ijk _is the observed phenotype, X_ijk _is a matrix of covariates, β_age _is the mean gradient of age (for all individuals), **A_i _**~ N(**0**, V_A_), **C_i _**~ N(**0**, V_C_), **Cs_i _**~ N(**0**, V_Cs_), **A.time_i _**~ N(**0**, V_A.time_), **C.time_i _**~ N(**0**, V_C.time_), **Cs.time_i _**~ N(**0**, V_Cs.time_), and E.time_ij _~ N(0, σ^2^_E.time_).

In this model time was measured using the covariate 'age'. The random effects for the gradient of age, A.time_ij_, C.time_ij_, Cs.time_ij_, and E.time_ij_, have a covariance structure equivalent to that described previously [1,2], with each variance component replaced by its counterpart for the gradient, so (for example) in this part of the model σ^2^_A _is represented by σ^2^_A.time_. However, the variance components for the intercept (σ^2^_A_, σ^2^_C_, σ^2^_Cs_, and σ^2^_E_) are independent of the variance components for the gradient (σ^2^_A.time_, σ^2^_C.time_, σ^2^_Cs.time_, and σ^2^_E.time_), and the estimated random effects for each variance component for the intercept are different from the estimated random effects for each variance component for the gradient. All random effects are constant across observations on the same individual; they do not change over time. Just as the size of σ^2^_A _can indicate whether additive genetic effects influence the outcome, the size of σ^2^_A.time _can indicate whether additive genetic effects influence the rate of change of the outcome with time.

All covariates were centered before inclusion in the model. The main model included the covariates male sex, cohort, height, drink, CPD, and some models also included BMI ((weight in kg)/(height in m)^2^) as a continuous variable. To allow for a nonlinear increase in SBP with age, quadratic and cubic age terms were also included.

The models were fitted using Gibbs sampling in WinBUGS v1.3 [3]. Vague normal N(0, 1000) priors were specified for fixed regression coefficients. Vague Pareto (1,0.001) priors were specified on the precisions (inverses of the variances) of all random effects; these were equivalent to specifying uniform priors on the corresponding variances. The priors for the intercept variance components (and associated random effects) were independent of the priors for the gradient variance components (and associated random effects). Models were run for 20,000 iterations after a burn-in of 5000 iterations. Although the model is Bayesian, the effect of each covariate was also assessed using a pseudo-likelihood approach.

### Blood pressure adjustment

The problem of how to model continuous SBP when some individuals are on blood pressure treatment is complex. As Cui et al. state, "the usual regression techniques used to adjust for the effects of antihypertensive treatment are inappropriate because they result in treated levels having an average of zero residuals rather than the extreme residuals they deserve, given their pretreatment pressures" [4]. Since complex modelling of SBP was not the main aim of this particular analysis, we chose a simple method of adjustment, which involved adding a constant (10 mm Hg) to each phenotype in which the individual was on treatment, to reflect the 'true' SBP that might have been observed if the individual had not been on treatment [4].

### Linkage analysis

As described previously [1,5] the random effects due to σ^2^_A _(A_ij_, the sigma-squared A residuals or SSARs) can be used as a continuous phenotype in a linkage analysis. The SSARs for the gradient (A.time_ij_, the sigma-squared A time residuals, or SSATRs) may also be used in such a way. The estimated SSARs and SSATRs were used as continuous phenotypes in a variance components linkage analysis that was performed using MERLIN [6]. All 399 markers were included.

## Results

### Variance components analysis

Results from fitting the longitudinal GLMM are shown in Table 1. Means ('best estimates') and 95% credible intervals are presented for each parameter. We deliberately excluded certain intermediate phenotypes (such as cholesterol) from the model even though they were clearly related to SBP, because they may lie on the causal pathway leading to high blood pressure and thus their inclusion in the model may produce misleading results. Many intermediate phenotypes also had few repeated measurements, so their inclusion would have greatly reduced the amount of available data. It is unclear whether BMI is an intermediate phenotype, but models including and excluding this covariate were very similar, and since measurements were available at almost all time points, results from the model which included BMI are presented in Table 1.

The size of σ^2^_A.time _(relative to the other variance components for the gradient) suggests that genes strongly influence the rate of change of SBP with age, while nongenetic influences due to shared familial or sibling environment are much less important. The same is true of the variance components for the intercept; genetic effects appear to account for the most variation in SBP at the mean age.

The mean rate of change of SBP with each year was 0.54, suggesting that for the average individual, SBP increases by approximately 5 mm Hg every 10 years. A nonlinear effect was also apparent, because the credible intervals for both the quadratic and cubic terms excluded 0. The 'average curve' for the relationship between BP and age (not included) showed an approximately linear relationship throughout most of the age range. When the quadratic and cubic terms were excluded most results changed little.

Other covariates also appeared to influence SBP. Males tended to have slightly lower SBP than females, and individuals from the second cohort tended to have slightly lower SBP than those included in the first cohort. The number of cigarettes smoked per day was also important, with an increase in BP of approximately 1 mm Hg for every six cigarettes. BMI was an important predictor of SBP, while height and number of drinks per day appeared to show little association with SBP.

Results changed little when σ^2^_E.time _was excluded, when σ^2^_C _and σ^2^_C.time _were excluded, when σ^2^_Cs _and σ^2^_Cs.time _were excluded, and when the SBP adjustment (for those on blood pressure treatment) was changed to +5 mm Hg or +20 mm Hg (instead of +10 mm Hg).

### Linkage results

Statistically significant linkage (LOD > 3, *p *< 0.00001) was found for both the SSARs and the SSATRs as shown in Table 2. The strongest results were for chromosome 21. Several other markers had LOD scores between 2 and 3; while these are not statistically significant at the genome-wide level they are regarded as 'possibly linked' and are also included in Table 2.

## Discussion

Our results corresponded well with the answers provided. The substantive conclusions of the variance components analysis were all consistent with the simulation model. However, in the linkage analysis, the SSARS appeared to detect both baseline and slope genes better than the SSATRs. Two simulated genes that affected SBP slope, as well as several genes which indirectly affected SBP through other phenotypes, were within 15 cM of our positive result on chromosome 21. A simulated gene on chromosome 19 also indirectly affected SBP. A gene for SBP slope was simulated on chromosome 15 (4 cM) and a gene for baseline SBP was simulated on chromosome 5 (176 cM), both quite close to the locations we found. Other genes that indirectly affected SBP were also nearby on these two chromosomes. However, our results for chromosome 4 and chromosome 6 were false positives.

Our methods have several advantages. They make full use of the extra information contained in the repeated observations for each individual, without first requiring the information to be summarized in some way. It is easy to include extra covariates or candidate gene information. They allow the inclusion of all pedigree members, without requiring decomposition of the pedigrees into smaller structures. We are currently working on extending the GLMMs to incorporate linkage directly, and this provides extra cohesion in these types of analysis. It also ensures that uncertainty in the estimates of the random effects is allowed for in the linkage analysis. However, the extra complexity in the unified models means that they can take considerably longer to fit.

Our methods had several features in common with others presented at GAW13. In particular, MacGregor et al. [7] and Yang et al. [8] used variance components models for longitudinal data, although their models also incorporated variance components for a QTL effect and they used maximum likelihood rather than MCMC fitting methods. Since they both analyzed the real data set, our results cannot directly be compared. Further comparisons of our method with those used by other authors may be found in the group summary paper by Gauderman et al. [9].

## Conclusions

The results we obtained suggest that our extended variance components models perform well in practice. They are relatively straightforward to fit, and produce useful and biologically plausible results.

## Figures and Tables

**Table 1:** Results from main GLMM

**Parameter**	**Mean**	**95% Credible Interval**
**Fixed effects**		
β_0_	134.4	(133.2, 135.5)
β.cohort2	-0.9846	(-1.694, -0.3023)
β.male	-0.9899	(-1.959, -0.08858)
β.hgt	1.739	(-3.319, 7.014)
β.bmi	0.1429	(0.07583, 0.208)
β.drink	0.005914	(-0.014, 0.02612)
β.cpd	0.1621	(0.1505, 0.174)
β.age	0.5445	(0.5167, 0.5736)
β.age2^A^	-0.002039	(-0.00248, -0.001599)
β.age3^B^	-4.48 × 10^-5^	(-6.49 × 10^-5^, -2.48 × 10^-5^)
**Variance components**		
σ^2^_A_	213.9	(199.8, 228)
σ^2^_C_	5.99	(0.389, 16.13)
σ^2^_Cs_	8.476	(4.271, 13.53)
σ^2^_E_	11.63	(11.4, 11.86)
σ^2^_A.time_	0.1273	(0.1088, 0.1436)
σ^2^_C.time_	0.007721	(1.83 × 10^-4^, 0.01722)
σ^2^_Cs.time_	0.01449	(0.006833, 0.02293)
σ^2^_E.time_	0.007635	(1.10 × 10^-4^, 0.01995)

^A^Quadratic term for age; ^B ^Cubic term for age.

**Table 2:** Summary of linkage results

**Chromosome**	**Phenotype**	**Marker**	**Location**	**LOD**	***p*-Value**
**LOD > 3**					
	21 SSAR	GATA188F04	40 cM	12.92	< 0.00001
	21 SSATR	GATA188F04	40 cM	5.10	< 0.00001
	19 SSAR	Mfd139	68 cM	3.88	0.00001
**2 < LOD < 3**					
	15 SSAR	GATA81F03	5 cM	2.99	0.00010
	6 SSAR	SE30	9 cM	2.86	0.00014
	5 SSAR	GATA6E05	160 cM	2.36	0.0005
	4 SSATR	GATA107	146 cM	2.21	0.0007
